# SuperSelective primer pairs for sensitive detection of rare somatic mutations

**DOI:** 10.1038/s41598-021-00920-4

**Published:** 2021-11-17

**Authors:** Fred Russell Kramer, Diana Yaneth Vargas

**Affiliations:** Public Health Research Institute, New Jersey Medical School, Rutgers University, Newark, NJ USA

**Keywords:** PCR-based techniques, Genetic mapping

## Abstract

SuperSelective primers, by virtue of their unique design, enable the selective exponential amplification of rare DNA fragments containing somatic mutations in the presence of abundant closely related wild-type DNA fragments. However, when a SuperSelective primer is used in conjunction with a conventional reverse primer, linear amplification of the abundant wild-type fragments occurs, and this may lead to a late arising signal that can be confused with the late arising signal from the rare mutant fragments. We have discovered that the use of a pair of SuperSelective primers, one specific for the target mutation in a plus strand, and the other specific for the same mutation in the complementary minus strand, but both possessing 3′-terminal nucleotides that are complementary to the mutation, significantly suppresses the linear amplification of the related wild-type sequence, and prevents the generation of false mutant sequences due to mis-incorporation by the DNA polymerase. As a consequence, the absence of mutant fragments in a sample does not give rise to a false-positive signal, and the presence of mutant fragments in a sample is clearly distinguishable as a true-positive signal. The use of SuperSelective primer pairs should enhance the sensitivity of multiplex PCR assays that identify and quantitate somatic mutations in liquid biopsies obtained from patients with cancer, thereby enabling the choice of a targeted therapy, the determination of its effectiveness over time, and the substitution of a more appropriate therapy as new mutations arise.

## Introduction

DNA fragments isolated from the plasma in a routine blood sample (a liquid biopsy) may transiently contain somatic mutations that arise in cancer cells, whose identity, if determined, can indicate which targeted therapy is likely to be effective against that patient’s cancer^1^. However, these DNA fragments, which originate from cancer cells that have died, are frequently quite rare, and are difficult to detect and quantitate because the plasma also contains abundant DNA fragments arising from normal cells that have died, including abundant closely related wild-type DNA fragments that often differ from the rare mutant DNA fragments by only a single-nucleotide variation^2^.

Our laboratory has been developing polymerase chain reaction (PCR) assays that utilize “SuperSelective primers” that are designed to selectively amplify rare mutant DNA fragments, without amplifying abundant closely related wild-type DNA fragments^3^. These primers are linear DNA molecules consisting of three sections: a relatively long “5′-anchor sequence” that enables the primer to bind to both mutant and wild-type DNA fragments containing the gene segment of interest; a short “3′-foot sequence” that is perfectly complementary to the segment of the mutant DNA fragment containing the somatic mutation, but mismatches the same segment in wild-type DNA fragments; and an intermediate-length “bridge sequence” that separates the anchor sequence from the foot sequence, and is chosen so that it is not complementary to any known human sequence. The sequence within the target DNA fragment to which the primer’s anchor sequence binds is separated from the sequence within the target DNA fragment to which the primer’s foot sequence binds by an “intervening sequence.” Consequently, by virtue of the single-stranded “bubble” formed by the bridge sequence in the primer and the intervening sequence in the DNA fragment, the robust gene-recognition function of the anchor hybrid is physically separated from the highly selective mutant recognition function of the short foot hybrid.

We believe that selection works as follows^3^. During the annealing stages of the PCR assay, the perfectly complementary short foot hybrids formed with the mutant DNA fragments last for hundreds of milliseconds before they fall apart, while the even shorter mismatched foot hybrids formed with the wild-type DNA fragments last for only a few milliseconds. Moreover, the Brownian motion of the assay solution impinging upon the single-stranded bubbles tends to rip the foot hybrids apart, and the mismatched wild-type foot hybrids are more easily ripped apart than the perfectly complementary mutant foot hybrids. Consequently, there is a significantly greater probability that a DNA polymerase will find and bind to a mutant foot hybrid and initiate exponential synthesis before that foot hybrid dissociates, than the considerably lower probability that a DNA polymerase will initiate synthesis on a very short-lived mismatched wild-type foot hybrid. Moreover, we have found that the inclusion of tetramethylammonium chloride (TMAC) in the PCR assay buffer tends to stabilize the perfectly complementary mutant foot hybrids, and tends to destabilize the mismatched wild-type foot hybrids, especially if the mismatch occurs at the 3′ nucleotide of the foot of the SuperSelective primer^4^.

Typically, these PCR assays are carried out with one SuperSelective primer and a conventional reverse primer for the amplification of rare DNA fragments possessing the target mutation, and the results enable those rare target molecules to be detected in the presence of abundant closely related wild-type molecules^3^. However, when ten or fewer target molecules are present in a sample, it is not clear whether the resultant late arising signal is due to the presence of those rare mutant target molecules (a true-positive signal), or whether the resultant late arising signal is due to the undesired amplification of the abundant closely related wild-type molecules (a false-positive signal). This uncertainty limits the desired sensitivity of the PCR assay. This situation arises because the conventional reverse primers initiate the generation of copies of the (+) strands of both the wild-type target fragments and the mutant target fragments during every thermal cycle of the PCR assay, even though the SuperSelective primers are designed to only generate copies of the resulting mutant (−) strands. For example, if there are 10,000 closely related wild-type fragments in the sample, then 10,000 additional wild-type (−) strands will be generated by the extension of the conventional reverse primes during each thermal cycle. After, say, 50 thermal cycles have been completed, this linear amplification will result in there being 500,000 copies of the wild-type (−) strands in the assay mixture. This enhanced abundance of wild-type (−) strands increases the probability that the SuperSelective primers will accidentally initiate synthesis on one of those linearly amplified wild-type (−) strands, leading to the generation of a false-positive signal. There is also a low probability that when the conventional reverse primers copy the wild-type (+) strands, that mis-incorporation will occur, introducing a nucleotide substitution in the resulting (−) strand, creating a mutant target sequence, even though the sample may not contain mutant DNA target fragments.

We have now overcome the limits to the sensitivity of these SuperSelective PCR assays by replacing the conventional reverse primers with SuperSelective primers. In these new PCR assays, there is a pair of SuperSelective primers for each of the mutant DNA targets that we wish to detect. The forward SuperSelective primer binds to the (−) mutant target strand, and its 3′ nucleotide is complementary to the mutant nucleotide in that strand; and the reverse SuperSelective primer binds to the (+) mutant target strand, and its 3′ nucleotide is complementary to the mutant nucleotide in that strand. Because these reactions do not contain conventional reverse primers that amplify the closely related wild-type DNA fragments, the wild-type sequences are not amplified during each thermal cycle, making it quite unlikely that a SuperSelective primer will initiate amplification on a closely related wild-type sequence.

To illustrate the enhanced sensitivity of PCR assays that utilize SuperSelective primer pairs, we compared assays containing SuperSelective forward primers and conventional reverse primers to otherwise identical assays containing SuperSelective primer pairs, the results of which document the suppression of false-positive signals when SuperSelective primer pairs are utilized. In addition, utilizing samples containing DNA fragments from 10,000 human genomes, we showed that assays containing SuperSelective primer pairs reliably provide true-positive signals in samples containing as little as five mutant DNA target fragments, yet do not produce false-positive signals when mutant DNA target fragments are not present in the sample.

## Results

### Advantage of utilizing SuperSelective primer pairs

**Figure 1:**
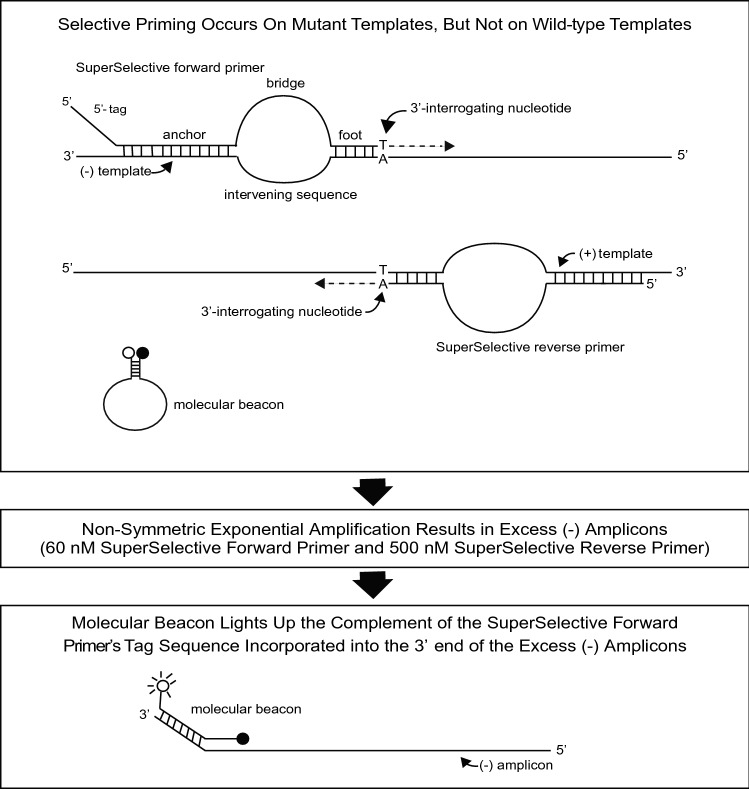
Design of PCR assays that utilize SuperSelective primer pairs for the selective amplification of rare mutant DNA target fragments in the presence of abundant DNA fragments from the entire human genome. After the SuperSelective primer pair initiates synthesis of amplicons on mutant target fragments (but does not initiate amplification on closely related wild-type DNA fragments), the resulting amplicons contain each primer’s bridge sequence in place of their target’s intervening sequence complement. Consequently, subsequent exponential amplification is highly efficient, since the entire sequence of each SuperSelective primer is complementary to the amplicons. Since the concentration of the forward primer is limited, and is eventually used up, the excess reverse primer continues linear amplification, creating readily available targets for molecular beacon probes, whose fluorescent color identifies their target amplicons.

To illustrate the enhanced selectivity and sensitivity of PCR assays that utilize SuperSelective primer pairs, we prepared samples that contained 10,000 copies of the entire human genome (including wild-type *KRAS* gene sequences) digested by incubation with restriction endonuclease MseI; and these samples also contained different amounts of a linearized plasmid containing a *KRAS* G12C mutant target sequence, in which an A:T base pair in the mutant *KRAS* sequence replaced a G:C base pair in the wild-type *KRAS* sequence. Figure 1 shows the design of these assays. Two SuperSelective primers are present: a forward primer that binds to the *KRAS* (−) sequence, whose 3′-terminal nucleotide is complementary to the mutant adenosine; and a reverse primer that binds to the *KRAS* (+) sequence, whose 3′-terminal nucleotide is complementary to the mutant thymidine. The forward primer also possessed a 5′-tag sequence (chosen so that it is not complementary to any known human sequence). In addition, these assays contained Quasar 670-labeled molecular beacon probes that were designed to bind to the complement of the 5′-tag sequence that is incorporated into the 3′ end of the resulting (−) amplicons, generating a fluorescent signal that reflects the amount of amplification that occurred. Furthermore, the concentration of the forward primer was limited (60 nM) and the concentration of the reverse primer was considerably greater (500 nM), so that after exponential amplification results in the incorporation of virtually all of the forward primers into (+) amplicons, the excess reverse primers continue to copy those (+) amplicons, generating an excess of (−) amplicons^5^. Consequently, the molecular beacon probes bind to those excess (−) amplicons with minimal competition from the limited (+) amplicons. These PCR assays were repeated, utilizing 500 nM of a conventional reverse primer in place of 500 nM of the SuperSelective reverse primer, in order to assess the advantage of utilizing SuperSelective primer pairs. The sequences of the primers and the molecular beacon probe used in these assays are shown in Table 1.

**Table 1:**
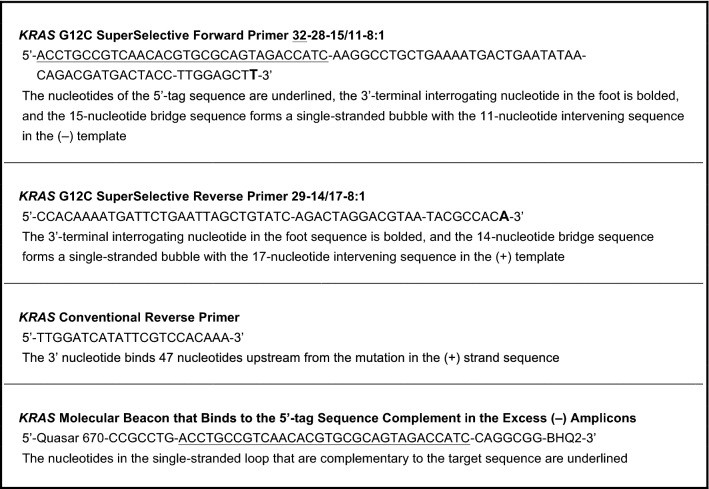
Primers and molecular beacon probe for the experiment that compares the use of a pair of SuperSelective primers to the use of a SuperSelective primer and a conventional reverse primer.

**Figure 2:**
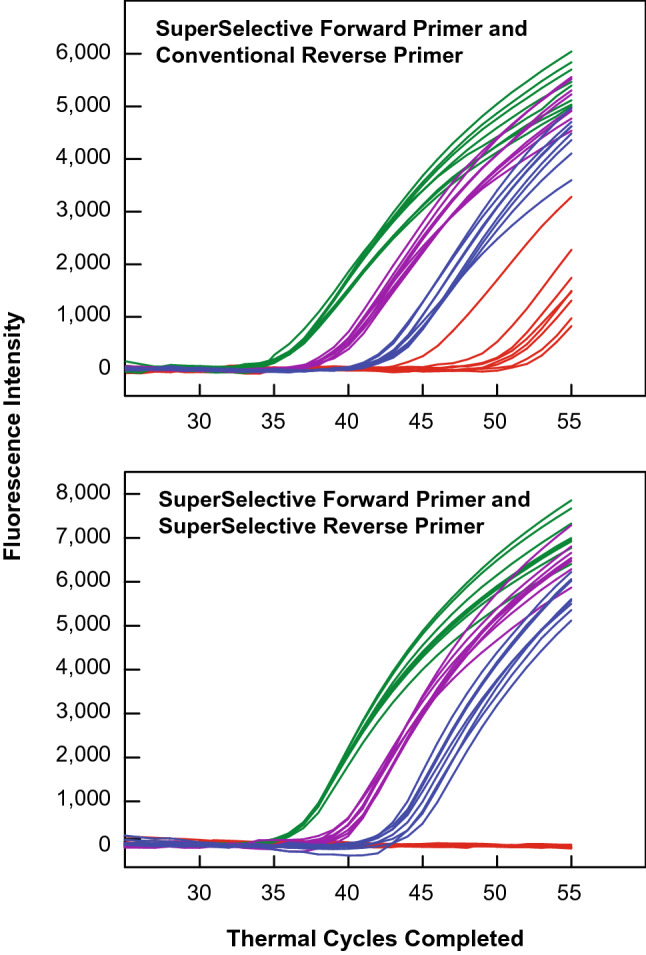
Demonstration of the selectivity of PCR assays that utilize a pair of SuperSelective primers for the amplification of rare mutant DNA fragments in the presence of abundant DNA fragments from the entire human genome. All of these reactions contained DNA fragments from 10,000 normal human genomes, including 10,000 copies of the wild-type *KRAS* sequence. In addition, these reactions contained different quantities of a linearized plasmid containing the *KRAS* G12C mutant target sequence: either 1,000 copies (shown in green), 100 copies (shown in purple), 10 copies (shown in blue), or 0 copies (shown in red). Each reaction was repeated eight times. The top panel shows the results that were obtained when only one of the primers for the *KRAS* mutant target was SuperSelective; and the bottom panel shows the results that were obtained when both primers for the *KRAS* mutant target were SuperSelective. When only one of the two primers was SuperSelective, false-positive signals arose when no *KRAS* mutant targets were present in the sample. When both primers were SuperSelective, the false-positive signals were suppressed.

Figure 2 shows the results of these assays. Every assay sample contained DNA restriction fragments from 10,000 human genomic DNA copies. In addition, the assay samples contained different amounts of linearized plasmids containing the *KRAS* G12C mutant sequence (either 1,000; 100; 10; or 0 copies). Furthermore, the assays initiated with each of the four sample types were repeated eight times.

The top panel shows the results obtained when these assays utilized a SuperSelective forward primer and a conventional reverse primer. The reactions that were initiated with either 1,000 or 100 mutant target molecules gave rise to signals that clearly occurred earlier than the signals arising from samples that only contained wild-type molecules. However, the signals arising from samples that contained only 10 mutant molecules, though occurring earlier than the signals arising from reactions that only contained wild-type molecules, could easily be confused with the signals arising from samples that only contained wild-type molecules; and as a practical matter the potential overlap of these true-positive signals with the false-positive background signals limits the sensitivity of these assays.

The bottom panel shows the results obtained when virtually identical assays were carried out that utilized a pair of SuperSelective primers, instead of a SuperSelective forward primer and a conventional reverse primer. The reactions that were initiated with either 1,000 or 100 or 10 mutant target molecules could easily be distinguished from each other, and the number of thermal cycles that were needed before a signal arose reflected the initial number of target molecules that were in each sample. Most importantly, signals did not arise from samples that only contained 10,000 wild-type molecules. Consequently, the use of SuperSelective primer pairs, instead of the use of a SuperSelective forward primer and a conventional reverse primer, suppresses the background to such an extent that the signal from ten mutant target molecules can confidently be considered to be a true-positive signal.

## Quantification of mutant abundance with improved sensitivity

To illustrate the extraordinary sensitivity of PCR assays that utilize SuperSelective primer pairs, we prepared samples that contained 10,000 copies of the entire human genome (including wild-type *EGFR* gene sequences and wild-type *β-actin* gene sequences); and these samples also contained different amounts of a linearized plasmid containing an *EGFR* G719C mutant target sequence, in which an A:T base pair in the mutant sequence replaced a G:C base pair in the wild-type *EGFR* sequence. These assays contained a pair of SuperSelective primers for the amplification of *EGFR* G719C target sequences, as well as a SuperSelective forward primer and a conventional reverse primer for the amplification of the *β-actin* reference gene (that when incorporated into an amplicon results in an interprimer region). In addition, these assays contained fluorescein-labeled molecular beacon probes that were designed to bind to the complement of the 5′-tag sequence that is incorporated into the 3′ end of the resulting mutant *EGFR* G719C (−) amplicons; and these assays contained Quasar 705-labeled molecular beacon probes that were designed to bind to the interprimer region of the resulting *β-actin* (−) amplicons. The signal from the amplified *β-actin* sequences served as an indicator of the amount of wild-type sequences that were present in the sample. The sequences of the primers and the molecular beacon probes used in these assays are shown in Table 2.

**Table 2:**
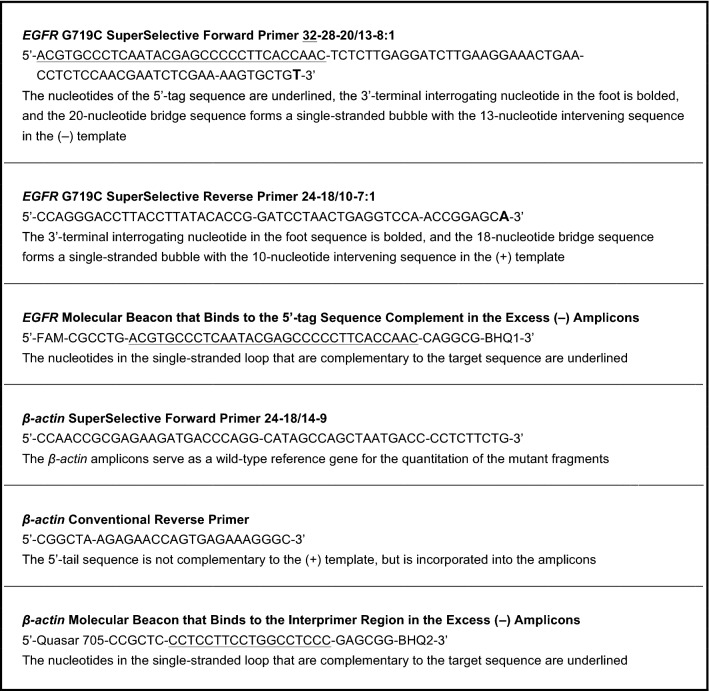
Primers and molecular beacon probes for the experiment that illustrates the selectivity and sensitivity of assays that utilize pairs of SuperSelective primers.

**Figure 3:**
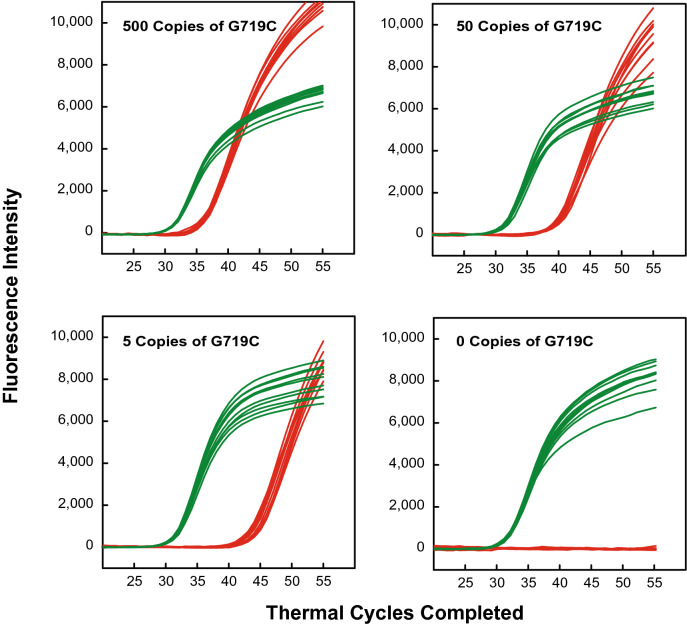
Demonstration of the sensitivity of PCR assays that utilize a pair of SuperSelective primers for the amplification of rare mutant DNA fragments in the presence of abundant DNA fragments from the entire human genome. Each reaction contained DNA fragments from 10,000 wild-type human genomes, including 10,000 copies of the *β-actin* reference gene (which reflects the amount of DNA in the sample). Four sets of ten identical PCR assays were carried out, each set containing either 500, 50, 5, or 0 copies of a linearized plasmid containing the *EGFR* G719C mutant DNA target sequence. Quasar 705-labeled molecular beacons lit up the amplicons generated from the *β-actin* reference gene (green curves), and fluorescein-labeled molecular beacons lit up the amplicons generated from the *EGFR* G719C mutant DNA target sequence (red curves). All ten reactions in which the sample contained only five mutant DNA target sequences produced a true-positive signal, while all ten reactions in which there were no *EGFR* G719C mutant DNA target sequences in the sample did not produce a signal above background.

Figure 3 shows the results of these assays. Four sets of assays were carried out, in which every sample contained DNA fragments from 10,000 wild-type human genomic DNA copies; and each of the four assay sets contained a different number of linearized plasmids containing the *EGFR* G719C mutant sequence (either 500; 50; 5; or 0 copies). Furthermore, every assay was repeated ten times. The resulting repeated amplification curves were quite similar. The fewer the number of *EGFR* G719C mutant DNA fragments that were present in the sample, the greater was the number of thermal cycles separating the *EGFR* G719C signal (fluorescein) from the *β-actin* signal (Quasar 705). The number of thermal cycles that separate the signal arising from the mutant fragments and the signal arising from the reference gene enable the abundance of the mutant sequences in the sample to be calculated, without needing to separately determine the actual amount of DNA in the sample^3^.

**Figure 4:**
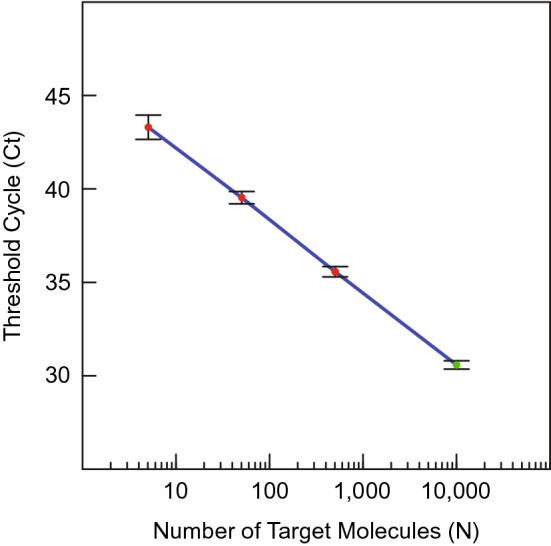
Inverse linear relationship between the mean Ct value of each set of *EGFR* G719C mutant DNA sequences (red dots) and the logarithm of the amount of those target DNA molecules present in each sample, including the mean Ct value of the *β-actin* reference gene fragments (green dot) contained in the 10,000 copies of the entire human genome. The standard deviation of each mean Ct value is indicated by vertical bars. The relatively larger standard deviation of the Ct values for samples that nominally contained five *EGFR* G719C mutant target DNAs reflects the Poisson distribution of these few template molecules among the ten reaction tubes.

These results also illustrate the quantitative relationship between the threshold cycle (Ct value) of each PCR assay and the actual number of DNA target sequences originally present in the sample (N). The mean Ct values of the reactions shown in Fig. 3, and the standard deviations of those Ct values, were: 30.57 ± 0.219 for 10,000 copies of the *β-actin* reference gene; 35.55 ± 0.284 for 500 copies of *EGFR* G719C mutant DNA; 39.51 ± 0.330 for 50 copies of *EGFR* G719C mutant DNA; and 43.27 ± 0.654 for 5 *EGFR* G719C mutant DNA. Figure 4 illustrates this relationship. The mean Ct values are inversely linearly proportional to the logarithm of the number of target DNA fragments originally present in the sample^6,7^. The equation of the line determined by a least squares fit of the data is: Log N = 11.925–0.259 Ct. Consequently, assays employing SuperSelective PCR primer pairs are able to determine the actual amount of mutant target DNAs in the sample, based on Ct values alone.

These results demonstrate that the use of SuperSelective primer pairs in assays for the detection and quantitation of rare mutant DNA fragments in the presence of abundant DNA fragments from the entire human genome can be accomplished with extraordinary sensitivity, due to the suppression of amplification of the abundant wild-type DNA fragments. True-positive signals arise, and false-positive signals are suppressed. When there were no *EGFR* G719C mutant DNA fragments in the samples, no fluorescein signals arose. Yet there was a distinct fluorescein signal when the samples contained as few as five *EGFR* G719C mutant DNA fragments.

## Discussion

PCR assays employing SuperSelective primer pairs and molecular beacon probes are rapid, low cost, and can be carried out on widely available spectrofluorometric thermal cyclers. Significantly, these PCR assays will be able to quantitate the abundance of somatic mutations in liquid biopsies obtained from cancer patients, enabling the choice of an effective targeted therapy, enabling a determination of the effectiveness of that therapy over time, and enabling the substitution of a different targeted therapy should new mutations arise^8^. In addition, these assays will be able to detect and quantitate minimal residual disease, so that potentially toxic therapies will only be recommended if the relative abundance of targeted mutant DNA fragments exceeds a predetermined clinically relevant level^9^. Moreover, rare antibiotic-resistant mutants can be detected in bacterial cultures from infected patients, enabling the choice of an effective antibiotic whose action is not compromised by the presence of the resistance mutation^10^. Furthermore, rare mutant paternal gene sequences can be detected in DNA fragments isolated from the blood of a pregnant women, potentially eliminating the need for an intrusive amniocentesis^11^. In summary, the enhanced sensitivity of multiplex PCR assays that employ SuperSelective primer pairs should enable a wide variety of effective new clinical diagnostic applications.

## Methods

### Wild-type human genomic DNA fragments

Genomic DNA (from multiple anonymous donors), catalog number G1521, was purchased from the Promega Corporation (Madison, WI). Approximately 9 µg of this DNA was digested for 120 min at 37 °C in 50 µL containing 10 Units of restriction endonuclease MseI (New England Biolabs, Ipswich, MA) in a buffer provided by New England Biolabs that contained 100 µg/mL bovine serum albumin, 10 mM magnesium acetate, 50 mM potassium acetate, and 20 mM Tris-acetate (pH 7.9); followed by incubation for 20 min at 65 °C to inactivate the enzyme. The resulting mixture of genomic DNA restriction fragments was analyzed by PCR to determine the concentration of the wild-type DNA fragments, after which the mixture was substantially diluted with water to create a stock solution containing a known number of closely related wild-type fragments/µL.

### Linearized plasmids containing target mutations

Plasmids containing the *KRAS* G12C mutant sequence were purchased from Integrated DNA Technologies (Coralville, IA), and were prepared by inserting a 200-base-pair sequence into pIDTSmart (Amp) vectors. These plasmids were linearized by digestion with 10 Units of restriction endonuclease MseI under the same conditions as described above for human genomic DNA. Plasmids containing the *EGFR* G719C mutant sequence were also purchased from Integrated DNA Technologies, and were prepared by inserting a 211-base-pair sequence into pUCIDT vectors. These plasmids were linearized by digestion with 10 Units of restriction endonuclease DraI (New England Biolabs) under the same conditions as described above for human genomic DNA. The concentration of each linearized plasmid was determined in a NanoDrop spectrophotometer (Thermo Fisher Scientific, Waltham, MA), after which the plasmids were diluted with water to create stock solutions containing known quantities of linearized target plasmids/µL.

### SuperSelective primers, conventional reverse primers, and molecular beacons

All primers were designed by us and were purchased from Integrated DNA Technologies. All molecular beacons were designed by us and were purchased from LGC Biosearch Technologies (Petaluma, CA). The concentration of each primer, and the concentration of each molecular beacon, was determined in a NanoDrop spectrophotometer.

### PCR assay components and procedures

All of the PCR assays were performed in 30-µL volumes that contained: 50 mM KCl, 2.5 mM MgCl_2_, 10 mM Tris-HCl (pH 8.0), 0.5% of the nonionic surfactant Tween 20 (Sigma-Aldrich, St. Louis, MO), 250 µM ATP, 250 µM CTP, 250 µM GTP, 250 µM TTP, and 1.5 Units of Platinum *Taq* DNA polymerase (Thermo Fisher Scientific).

The *KRAS* G12C assays also contained 50 mM tetramethylammonium chloride (Sigma-Aldrich), 60 nM *KRAS* G12C SuperSelective forward primer 32-28-15/11-8:1, either 500 nM *KRAS* conventional reverse primer or 500 nM *KRAS* SuperSelective reverse primer 29-14/17-8:1, and 200 nM of the Quasar 670-labeled *KRAS* molecular beacon.

The *EGFR* G719C assays also contained 60 mM tetramethylammonium chloride, 60 nM *EGFR* G719C SuperSelective forward primer 32-28-20/13-8:1, 500 nM *EGFR* G719C SuperSelective reverse primer 24-18/10-7:1, 300 nM of the fluorescein-labeled *EGFR* molecular beacon, 60 nM *β-actin* SuperSelective forward primer 24-18/14-9, 500 nM *β-actin* conventional reverse primer, and 500 nM of the Quasar 705-labeled interprimer-specific *β-actin* molecular beacon.

The PCR amplifications were carried out in 0.2 mL white polypropylene tubes (USA Scientific, Ocala, FL) in a CFX-96 Touch spectrofluorometric thermal cycler (Bio-Rad Laboratories, Hercules, CA). The thermal cycling program was 2 min at 95 °C, followed by 55 cycles of 95 °C for 20 s, 60 °C for 20 s, and 72 °C for 20 s. Molecular beacon fluorescence intensities were measured at the end of each 60 °C annealing stage. The number of thermal cycles that occurred before the intensity of each colored signal reached a predetermined level (the threshold cycle, Ct) was determined automatically by the spectrofluorometric thermal cycler.
